# The implementation of whole-genome sequencing for *Mycobacterium tuberculosis* in Vietnam

**DOI:** 10.5588/ijtldopen.24.0147

**Published:** 2024-07-01

**Authors:** D.T. Huong, T.M. Walker, D.T. Ha, K.T.T. Ngoc, V.N. Trung, L.T. Nam, P.T.T. Ngoc, L.T. Nguyet, N.T. Thanh, N.H. Minh, N.K. Cuong, N.V. Khiem, H.V.T. Ngoc, T.T.T. Bich, H.N. Hong, P.P. Trieu, L.K. Lan, K. Lan, N.N. Hue, N.T.L. Huong, T.L.T.N. Thao, N.L. Quang, T.D.D. Anh, D.W. Crook, G.E. Thwaites, N.T.T. Thuong, N.B. Hoa, D.V. Luong, N.V. Hung

**Affiliations:** ^1^ National Lung Hospital, Hanoi, Vietnam;; ^2^ Oxford University Clinical Research Unit, Ho Chi Minh City, Vietnam;; ^3^ UK Centre for Tropical Medicine and Global Health, Nuffield Department of Medicine, University of Oxford, Oxford, UK;; ^4^ Hanoi Lung Hospital, Hanoi, Vietnam;; ^5^ Nuffield Department of Medicine, University of Oxford, Oxford, UK;; ^6^ VNU Hanoi, University of Medicine and Pharmacy, Hanoi, Vietnam

**Keywords:** WGS, drug-resistant TB, MDR/RR-TB, extensively drug-resistant, capacity building

Dear Editor,

TB remains a major infectious disease with an estimated 10.6 million cases in 2022.^1^ Drug-resistant TB, particularly multidrug-resistant/rifampicin-resistant TB (MDR/RR-TB), remains a public health threat, accounting for 3.3% of new TB cases and 17% of previously treated cases worldwide. To curb the spread of TB drug resistance, accurate diagnosis and treatment are key, alongside epidemiological surveillance to inform community-level public health interventions. Molecular WHO-recommended rapid diagnostics (mWRDs) such as Xpert^®^ Ultra (Cepheid, Sunnyvale, CA, USA), and line-probe assays (LPAs) only detect resistance-conferring mutations in specific target regions and for a limited number of drugs.^2^ Whole-genome sequencing (WGS) can be used to tailor treatment plans for TB patients and inform surveillance. The feasibility of using WGS for the routine diagnosis of DR-TB has been demonstrated in high-income countries,^3^ but few data are available from low-income, high TB burden settings. Here, we present our experience of setting up WGS for *Mycobacterium tuberculosis complex* (MTBc) in Vietnam, describing the challenges and lessons learnt for other TB programmes that are planning to deploy WGS in similar settings.

Vietnam has an estimated TB incidence of 176/100,000 population with approximately 9,200 MDR-TB cases in 2022.^4^ MDR-TB accounts for 3.3% of new diagnoses and 15% of previously treated TB cases, but only 3,577 of an estimated 9,200 MDR-TB cases were laboratory-confirmed. The Vietnam National TB Control Programme (Vietnam NTP) guidelines recommend routine drug resistance detection using Xpert, Truenat (Molbio Diagnostics, Goa, India), and LPA. A research grant enabled the Vietnam NTP to work towards the implementation of WGS in the National TB Reference Laboratory (NTRL), Hanoi, Vietnam, with support from Oxford University Clinical Research Unit (OUCRU) in Ho Chi Minh City. The focus of this work was to implement technology to predict drug susceptibility for isolates from patients with RR-TB. The project coincided with the COVID-19 pandemic, which resulted in 11,522,432 cases in Vietnam, including 43,151 deaths between April 2021 and December 2022.^5^

The project was implemented over 3 years from May 2020 to April 2023. Before the first batch of sequencing was independently performed by NTRL staff, took a year of preparation, including equipment, reagents procurement, lab infrastructure innovation and training activities (Table). Foreseen fixed start-up costs included laboratory renovation, infrastructure upgrades, and capital expenditures, including the procurement of an Illumina MiniSeq platform (Illumina, San Diego, CA, USA), estimated at USD122,907. One clinical microbiologist and three laboratory technicians were trained as WGS master users in four training courses provided by OUCRU Ho Chi Minh (HCM) or Illumina’s specialists. The marginal costs of sequencing a batch of eight samples were composed of reagent and consumable costs for DNA extraction and library preparation, coming to USD1,307 (USD163 per sample). Our costs were comparable to those of the project in Kyrgyz Republic,^6^ whose reported costs ranged from USD141–167 per isolate on the Miseq (Illumina) run. Costs are generally lower in high-income countries, where the market for WGS is larger and can be further reduced by including more samples in a run.^7^

**Table.** Summary of the implementation of WGS for MTB at the Vietnam National TB Reference Laboratory.

No	Contents and descriptions	
1.	Sequencing platform:	
	Miniseq Illumina platform – whole-genome sequencing	
2.	Timeline:	
	A research grant-based implementation in 2020–2022 during COVID-19 outbreak which led to delays 2020 for preparation step 2021–2022 for sample collection, sequencing and data analysis	
4.	Cost:	
	Foreseen fixed start-up: USD122,907 Cost per sequenced sample: USD163	
4.	Human resources and training:	
	1 clinical microbiologist and 3 laboratory technicians who had a basic background in medical laboratory sciences and more than 4 years’ experience in TB diagnostics. Total 5 training courses provided: 1 for basic principles of NGS (3 days), 2 for DNA extraction, library preparation and sequencing (4 days each in OUCRU and NTRL), 1 for bioinformatics (1 day). Remote technical support via Zoom call, chat provided by OUCRU when necessary	
5.	Major technical challenges	Further solutions
	The complexity of WGS protocol and workflow	Received close technical support from OUCRU HCM team to set-up a sequencing laboratory and build staff capacity
	Disrupted online pipeline to predict drug resistance	Implemented the Clockwork pipeline on a specially purchased local high-performance computer
	Some sequencing running errors were caused by improper placement of the sequencing device.	Identified a separate and better temperature and humidity-controlled place for sequencing devices
	Challenges with storing the large amounts of data from WGS	Established a local server for data sequencing storage
6.	Future plan	
	Training more staff to mitigate against employee turnover Improving bioinformatics skills to make best use of the sequencing data Procuring another sequencing platform with a larger capacity and a freezer with back-up power supply Participating in a proficiency testing programme	

WGS = whole-genome sequencing; MTB = *M. tuberculosis*; NGS = next-generation sequencing; OUCRU = Oxford University Clinical Research Unit; HCM = Ho Chi Minh.

MTBc isolates from adult patients with RR-TB were collected and WGS was performed at NTRL using an Illumina MiniSeq platform. The Clockwork pipeline^8^ generated for the CRyPTIC Consortium (University of Oxford, Oxford, UK) was used to analyse FASTQ files, and the WHO catalogue of mutations in MTBc associated with drug resistance was used to make predictions around susceptibility.^9,10^

Because the project was interrupted by the COVID-19 pandemic, only 265 isolates were sequenced in total and the results are presented in a separate paper.^11^ Our focus here is on the challenges and lessons learnt. Most of the personnel at the National Lung Hospital were reallocated to COVID-19-related activities and the collection of MDR-TB samples was delayed because patients were unable to access healthcare services. Delays meant that some reagents expired or remained unused. We initially installed the MiniSeq sequencer in our DNA mixing and PCR room where routine testing was also being undertaken but soon encountered repeated MiniSeq running errors. The most likely cause was excessive humidity,^12^ and fluctuations in room temperature (as staff frequently entered and exited the air-conditioned room). The final challenge was data handling and analysis. The OUCRU team provided NTRL staff with training on how to use Oxford University’s cloud-based pipeline to predict drug resistance. However, during implementation, this service was discontinued (as funding ended) and a replacement was delayed. As a solution, NTRL implemented the Clockwork pipeline^8^ on a specially purchased local high-performance computer. This took considerable effort from the local team to set up, using online sources as a guide. We considered using Mykrobe (European Bioinformatics Institute, Cambridgeshire, UK),^13^ because it has an easy-to-use drag-and-drop interface, but we wanted access to all the data on resistance-associated genes relevant to all drugs in use, so opted against this.

Detection of MDR-TB and extensively drug-resistant (XDR-TB) in Vietnam still relies on mWRDs. The benefit of WGS is it provides more detailed data on resistance and susceptibility for a greater number of drugs, and more quickly than pDST, while also generating useful data on the control of transmission. However, there are challenges around the feasibility of implementing WGS for MTBc in low- and middle-income countries. Although others may have had different experiences, the lessons we have learned may be of value to settings that have yet to implement WGS, but are considering doing so. The next steps for us to effectively implement WGS include the following. First, we urgently need to identify a separate, better temperature-controlled room for the MiniSeq. We also need to train additional staff to mitigate against staff turnover. In terms of hardware, we need to establish a local server for data storage (alongside a backup solution), a Miseq platform with a larger capacity, and a freezer with backup power supply to increase the number of samples sequenced per batch and ensure the quality of the reagents. An external quality assurance programme will be required for full-scale clinical implementation. We plan to allocate financial support from our NTP’s partners to cover the cost of these activities. Finally, we need to improve our bioinformatics skills to maximise the use of the data that is generated, including for outbreak analysis and surveillance. This can be achieved by capacity building activities in collaboration with OUCRU.

The Vietnam NTP is planning to invest in and adopt WGS for DR-TB management and surveillance. WGS will thus soon be performed at the NTRL as recommended,^14^ and we aim to have enough capacity, infrastructure and personnel to perform high-quality sequencing of samples referred to us from across Vietnam.
